# metabolic profiling of Parkinson's disease and mild cognitive impairment

**DOI:** 10.1002/mds.26992

**Published:** 2017-04-10

**Authors:** Florence Burté, David Houghton, Hannah Lowes, Angela Pyle, Sarah Nesbitt, Alison Yarnall, Patrick Yu‐Wai‐Man, David J. Burn, Mauro Santibanez‐Koref, Gavin Hudson

**Affiliations:** ^1^ Mitochondrial Research GroupNewcastle UniversityNewcastle Upon TyneUK; ^2^ Institute for Cell and Molecular BioscienceNewcastle UniversityNewcastle Upon TyneUK; ^3^ Institute of NeuroscienceNewcastle UniversityNewcastle Upon TyneUK

**Keywords:** Parkinson's Disease, fatty acid beta oxidation, metabolomics

## Abstract

**Background**: Early diagnosis of Parkinson's disease and mild cognitive impairment is important to enable prompt treatment and improve patient welfare, yet no standard diagnostic test is available. Metabolomics is a powerful tool used to elucidate disease mechanisms and identify potential biomarkers.

**Objectives**: The objective of this study was to use metabolic profiling to understand the pathoetiology of Parkinson's disease and to identify potential disease biomarkers.

**Methods**: This study compared the serological metabolomic profiles of early‐stage Parkinson's patients (diagnosed < 12 months) to asymptomatic matched controls using an established array based detection system (DiscoveryHD4™, Metabolon, UK), correlating metabolite levels to clinical measurements of cognitive impairment.

**Results**: A total of 1434 serological metabolites were assessed in early‐stage Parkinson's disease cases (n = 41) and asymptomatic matched controls (n = 40). Post–quality control, statistical analysis identified n = 20 metabolites, predominantly metabolites of the fatty acid oxidation pathway, associated with Parkinson's disease and mild cognitive impairment. Receiver operator curve assessment confirmed that the nine fatty acid oxidation metabolites had good predictive accuracy (area under curve = 0.857) for early‐stage Parkinson's disease and mild cognitive impairment (area under curve = 0.759).

**Conclusions**: Our study indicates that fatty acid oxidation may be an important component in the pathophysiology of Parkinson's disease and may have potential as a diagnostic biomarker for disease onset and mild cognitive impairment. © 2017 The Authors. Movement Disorders published by Wiley Periodicals, Inc. on behalf of International Parkinson and Movement Disorder Society.

Early clinical diagnosis of Parkinson's disease (PD) is important in reducing symptoms, slowing disease progression, and improving patient welfare.^1^ Postmortem, PD is confirmed by the presence of Lewy bodies.^2^ In life, PD diagnosis is based on clinical observation of cardinal features^2^; however, the pathological processes leading to PD can begin decades before the actual symptoms begin, which can be mild in the early stages of disease. Imaging technologies have improved diagnosis; however, translating these into sensitive and specific predictors of individual neurodegenerative predisposition is challenging and, as of yet, there is no single blood‐based biomarker that can be used to identify early‐stage PD in clinical practice. Moreover, the majority of PD patients will go on to develop cognitive impairment and as of yet there is no predictive tool to identify at‐risk individuals.^3^

Metabolomics is a dynamic field that can be applied to disease pathogenesis to elucidate mechanisms and identify potential biomarkers.^4^ In recent years, there has been a growing interest in the use of metabolomics in PD research.^5^, ^6^, ^7^, ^8^, ^9^, ^10^, ^11^ However, many of these studies do not corroborate each other, possibly limited by low sample number and confounded by clinical heterogeneity and analytical methodology.^6^

Here we report comprehensive serological metabolomic profiling using a well characterized cohort of early‐stage PD patients and matched controls with the aim of identifying potential biomarkers for the onset of PD and to investigate the pathophysiological changes associated with disease.

## Methods

Fasting serum samples from 41 idiopathic early‐stage Parkinson's patients (disease duration < 1 year, mean = 5.4 months, standard deviation = 4.9 months) and 40 age‐ and gender‐matched controls were selected from the Incidence of Cognitive Impairment in Cohorts with Longitudinal Evaluation – Parkinson's Disease (ICICLE‐PD) study^12^ and included for metabolomic profiling. All PD cases were assessed locally by a movement disorder specialist using Queen Square Criteria for the diagnosis of PD^13^ and were treatment naïve. Summary demographic data is shown in Supplementary Table 1.

All PD cases and age‐matched controls underwent cognitive assessment. Global cognitive function was assessed using the Mini‐Mental State Examination (MMSE)^14^ and the Montreal Cognitive Assessment (MoCA).^15^ Mild cognitive impairment (MCI), assessed during ICICLE‐PD,^12^ was determined using published criteria (using 1.5 SD as a cut‐off) and all individuals were stratified as MCI‐yes or ‐no.^12^ Summary clinical data is shown in Supplementary Table 1. Serum brain derived neurotrophic factor (BDNF) levels were measured by an established sandwich enzyme‐linked immunosorbent assay (ELISA) kit (Promega, Sweden, Stockholm) as per the manufacturer's guidelines.

Nontargeted mass spectrometry‐based metabolomic profiling (n = 1434 biochemicals) was performed on all 81 serum samples using the established DiscoveryHD4™ Metabolon platform (Metabolon, Cambridge, UK), described previously.^11^, ^16^, ^17^, ^18^ Metabolomic profiling was performed in case‐control randomized batches to control for run‐specific variation. Previously described Quality control (QC) was used.^16^, ^17^, ^18^ Briefly serum samples with >10% missing data points (n = 0 case and n = 0 control) were excluded from further analysis, and metabolites with >10% missing data points through limited detection (n = 43) were also removed from further analysis.

### Statistical Analysis

Raw metabolite values are normalized by range scaling, setting the median equal to 1.^19^ Normalized data were analyzed using SPSS (Version 22.0. Armonk, NY: IBM Corp.) with data appropriate tests (detailed in text). Statistical significance was set at *P* < .05 after Bonferroni correction for multiple testing. Principal component analysis (PCA) was performed using the FactoMineR (v1.24) package in R (v3.3.1). Orthogonal Projections to Latent Structures Discriminant Analysis (OPLS‐DA) plots and associated analysis were generated using MetaboAnalyst 3.0.^20^

### Ethical Approval and Consent to Participate

Serological samples were obtained with consent from Local Research Ethics Committee (LREC) (reference 08/H0906/147).

## Results

**Figure 1:**
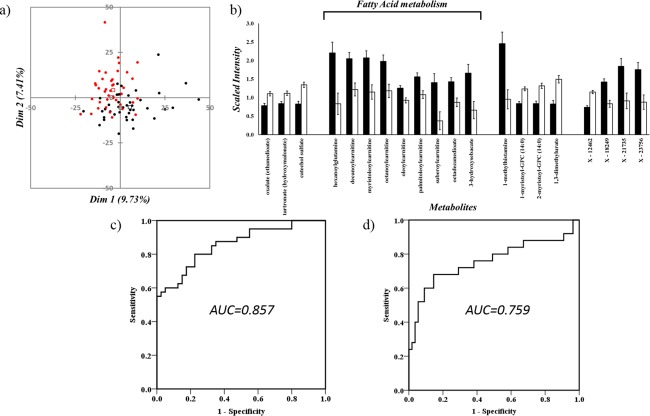
**a**: Score plots of Principle Component 1 (PC1) versus Principle Component 2 (PC2) from principal component analysis (PCA) of post‐QC scaled metabolite data (n = 1393) in cases (red) and controls (black), showing corresponding centers of gravity (open squares). **b**: Comparative mean scaled intensities for the n = 20 significantly different metabolites between early‐stage PD (shaded) and age‐matched controls (unshaded), showing standard error of the mean. Highlighted are the nine metabolites associated with fatty acid beta oxidation. **c**: ROC curve of a logistic regression model for distinguishing early stage PD from matched controls using the 9 fatty acid beta oxidation (FAO) metabolites (where AUC is area under the curve). **d**: ROC curve of a logistic regression model for distinguishing MCI in early‐stage PD from cognitively normal early‐stage PD (Mild‐cognitive‐impairment‐normal MCI‐n); where AUC is area under the curve). [Color figure can be viewed at wileyonlinelibrary.com]

PCA of post‐QC scaled metabolite data (n = 1393) indicates that early‐stage PD cases and matched controls have distinct metabolic profiles (Fig. 1a), supported by separation using cross‐validated orthogonal partial least squares discriminant analysis (OPLS‐DA, R2 = 0.77 and Q2 = 0.50; Supplementary Fig. 1).^21^ Mann–Whitney *U* testing, after Bonferroni correction for multiple testing, identified 20 metabolites that were significantly different between early‐stage PD cases and matched controls; 13 were significantly increased and 7 significantly decreased in cases (Fig. 1b and Table 1). Subsequent annotation through the Kyoto Encyclopaedia of Genes and Genomes database revealed a consistent and significant increase of 9 metabolites of the fatty acid beta oxidation (FAO) pathway, including components of both the acyl glutamine and acylcarnitine pathways: hexanoylglutamine, decanoylcarnitine, myristoleoylcarnitine, octanoylcarnitine, oleoylcarnitine, palmitoleoylcarnitine, suberoylcarnitine, octadecanedioate, and 3‐hydroxysebacate. In addition, 1‐methylhistamine, a component of histidine metabolism, and 3 xenobiotics (Metabolon ID, x–18249, x–21735, and x‐23756) were significantly increased in PD. Because age and gender are predictors of PD^22^ and, given the strong association to fatty acid metabolism, we confirmed each association through multivariate analysis, with age, gender, and body mass index as covariates (Table 1). Kyoto Encyclopaedia of Genes and Genomes annotation of the 7 significantly reduced metabolites identified the following 4 pathways: ascorbate/aldarate metabolism, benzoate metabolism and lysolipid, xanthine metabolism, which remained significant after multivariate analysis with age and gender as covariates (Table 1).

**Table 1:** Post–Bonferroni corrected association results showing the 20 metabolites associated with early‐stage PD

Compound	Canonical pathway	Mean PD	SEM	Mean control	SEM	Mann–Whitney *U* a	Binary logistic regression^b^	ROC AUC	MCI	BDNF
Oxalate (ethanedioate)	Ascorbate and aldarate metabolism	0.784	0.060	1.105	0.060	8.00 × 10^−03^	1.81 × 10^−03^	0.756	9.5 × 10^−02^	2.64 × 10^−01^
Tartronate (hydroxymalonate)	Bacterial/fungal	0.841	0.055	1.121	0.056	1.50 × 10^−02^	2.28 × 10^−03^	0.749	2.6 × 10^−02^	2.61 × 10^−01^
Catechol sulfate	Benzoate metabolism	0.831	0.068	1.344	0.073	4.00 × 10^−03^	2.48 × 10^−04^	0.767	1.3 × 10^−01^	9.17 × 10^−04^
Hexanoylglutamine	Fatty acid metabolism (acyl glutamine)	2.208	0.285	0.836	0.289	8.00 × 10^−04^	1.14 × 10^−03^	0.790	2.0 × 10^−03^	2.43 × 10^−04^
Decanoylcarnitine	Fatty acid metabolism (acyl carnitine)	2.050	0.169	1.221	0.177	2.30 × 10^−03^	3.10 × 10^−04^	0.775	5.0 × 10^−03^	1.07 × 10^−02^
Myristoleoylcarnitine	Fatty acid metabolism (acyl carnitine)	2.076	0.189	1.151	0.196	5.10 × 10^−03^	9.61 × 10^−04^	0.763	4.4 × 10^−02^	3.03 × 10^−04^
Octanoylcarnitine	Fatty acid metabolism (acyl carnitine)	1.978	0.173	1.184	0.180	6.80 × 10^−03^	3.96 × 10^−04^	0.759	1.8 × 10^−02^	1.13 × 10^−03^
Oleoylcarnitine	Fatty acid metabolism (acyl carnitine)	1.257	0.069	0.929	0.070	2.56 × 10^−02^	1.72 × 10^−03^	0.737	1.0 × 10^−03^	6.70 × 10^−03^
Palmitoleoylcarnitine	Fatty acid metabolism (acyl carnitine)	1.563	0.102	1.081	0.104	5.51 × 10^−02^	1.29 × 10^−03^	0.724	1.2 × 10^−03^	5.22 × 10^−03^
Suberoylcarnitine	Fatty acid metabolism (acyl carnitine)	1.407	0.239	0.377	0.242	2.00 × 10^−04^	1.13 × 10^−03^	0.796	1.0 × 10^−03^	5.27 × 10^−03^
Octadecanedioate	Fatty acid, dicarboxylate	1.427	0.113	0.873	0.118	4.70 × 10^−03^	1.33 × 10^−03^	0.764	6.3 × 10^−02^	5.10 × 10^−03^
3‐hydroxysebacate	Fatty acid, monohydroxy	1.663	0.231	0.662	0.233	8.74 × 10^−05^	4.50 × 10^−04^	0.819	5.0 × 10^−03^	3.51 × 10^−02^
1‐methylhistamine	Histidine metabolism	2.456	0.312	0.955	0.257	4.00 × 10^−03^	9.79 × 10^−04^	0.767	2.1 × 10^−01^	2.23 × 10^−03^
1‐myristoyl‐GPC (14:0)	Lysolipid	0.843	0.054	1.237	0.054	5.10 × 10^−03^	5.28 × 10^−04^	0.763	2.7 × 10^−02^	1.16 × 10^−01^
2‐myristoyl‐GPC (14:0)	Lysolipid	0.840	0.069	1.319	0.071	3.50 × 10^−03^	5.66 × 10^−04^	0.769	1.1 × 10^−01^	1.06 × 10^−01^
1,3‐dimethylurate	Xanthine metabolism	0.830	0.096	1.496	0.098	3.60 × 10^−03^	6.55 × 10^−04^	0.768	7.4 × 10^−02^	8.70 × 10^−01^
x ‐ 12462	Unknown	0.742	0.044	1.152	0.047	4.00 × 10^‐04^	9.07 × 10^−04^	0.800	7.3 × 10^−02^	1.38 × 10^−03^
x ‐ 18249	Unknown	1.421	0.088	0.835	0.092	5.10 × 10^‐03^	8.40 × 10^−05^	0.848	3.4 × 10^−01^	1.74 × 10^−03^
x ‐ 21735	Unknown	1.847	0.212	0.912	0.211	1.10 × 10^‐02^	5.74 × 10^−04^	0.751	7.3 × 10^−02^	1.38 × 10^−02^
x ‐ 23756	Unknown	1.754	0.190	0.880	0.194	8.60 × 10^‐03^	1.30 × 10^−03^	0.755	3.5 × 10^−01^	2.39 × 10^−02^

Shown are the normalized mean metabolite levels in PD cases and matched controls, standard error of the mean (SEM), case/control comparison by Mann–Whitney *U* testing, binary logistic regression (with age, gender, and body mass index—fatty acid metabolism only—as covariates), and receiver operator area under the curve (ROC AUC) assessments of predictive ability. In addition the uncorrected significance of Mann–Whitney *U* test of mild cognitive impairment (MCI, stratified as y/n) and the bivariate Pearson's correlation coefficient probability to serological brain derived neurotrophic factor (BDNF) levels are shown.

a Mann–Whitney *U* testing.

b Binary logistic regression.

To assess the ability of each metabolite to determine PD status, receiver operator curves (ROC) were generated. All previously significantly different metabolites showed individual areas under the curve (AUC) > 0.70 (Table 1), indicative of good predictive accuracy.^23^ Furthermore, a combined ROC curve, constructed using binary logistic regression^8^, ^9^ of the metabolically linked FAO metabolites (n = 9, Table 1) indicated increased predictive accuracy (AUC = 0.857) for early‐stage PD patients (Fig. 1c).

The measurements of cognitive decline in early‐stage PD are well documented,^12^ and we identified significant associations (Bonferroni‐corrected Mann–Whitney *U, P* < .05), particularly to metabolites involved in FAO, when stratifying PD cases by mild cognitive impairment (MCI‐y) versus cognitively normal (MCI‐n; Table 1), with cognitively impaired PD cases having significantly higher levels of FAO metabolites than cognitively normal cases (Supplementary Table 2). Similar to PD, a combined ROC curve of the 9 FAO metabolites indicated good predictive accuracy for MCI in early‐stage PD patients (AUC = 0.759, Fig. 1d). However, despite a priori disease association to MMSE and MoCA (Supplementary Table 1), we were unable to correlate either to significant metabolite levels (Supplementary Table 3).

Serological levels of brain‐derived neurotrophic factor (BDNF), a neuroprotective member of the trophin family, have been linked to PD.^24^, ^25^ With this in mind, we correlated the significantly different metabolites in this study to serological BDNF levels in our cohort, identifying a significant correlations (Pearson's correlation ranges *r* = 0.33‐0.39) between increased metabolites of FAO and increasing BDNF (Table 1).

## Discussion

Our metabolomic data suggest that several metabolites, particularly carnitines of the FAO pathway, are highly associated with the onset of PD and correlate with MCI and the expression of the neuroprotective factor BDNF.

Dysregulation of FAO, typically an enzymatic deficiency in either fatty acid breakdown, such as in medium‐chain acyl‐CoA dehydrogenase deficiency, or disruption of fatty acid transport across the mitochondrial membrane through defects in the carnitine transport system, primarily results in a metabolic disorder with fatty acids reaching cytotoxic levels.^26^ Our study of early‐stage PD serum showed a significant increase of the metabolic intermediates of FAO when compared with controls, an indicator that FAO is actually increased. This is supported by 2 previous metabolic studies^8^, ^9^ that identified a significant increase in PD patient urinary furoylglycine, triglylglycine and hexanoylglycine, which are components involved in mitochondrial fatty acid β‐oxidation.^8^, ^9^, ^27^

When oxygen supply cannot meet demand, that is, during neuronal activation or the development of pathology, the brain shifts from glucose metabolism to inefficient anaerobic respiration.^28^ However, the brain also has the capacity to shift energy production from glucose metabolism entirely, using FAO or ketones during pathological conditions such as neurodegeneration, hypoxia/ischemia, or posttraumatic brain injury.^29^ It is therefore possible that the increase in FAO we observed in early‐stage PD cases is an attempt at attenuating neuronal cell death, a hypothesis supported by the upregulation of BDNF, a neuroprotective agent and further supported by our observed significant increase in 1‐methylhistamine, a metabolite of histamine metabolism. Histamine has a role in neuronal transmission,^30^ and elevated metabolite levels have been observed in schizophrenia,^31^ Alzheimer's disease, and PD patients.^32^ Similar to BDNF, increased histamine is a marker of neuronal damage,^32^ and our data suggest that 1‐methylhistamine could indeed be an additional marker for neurodegeneration in early‐stage PD.

In addition, 7 metabolites were significantly reduced in PD cases when compared with controls, supportive of earlier observations in PD. Reduced ascorbate/aldarate metabolism, a marker of vitamin C deficiency, was detected at subclinical levels in a previous study in early PD.^33^ Catechol sulfate, a metabolite of benzoate metabolism, is a product of both liver/kidney function and gut microflora metabolism, and a decrease may be indicative of changes in PD gut microbiota.^34^ Xanthine metabolism and lysolipid metabolism were both reduced in our early‐stage PD cases, characterized by a reduction in 1‐myristoyl‐glycero‐3‐phosphocholin, 2‐myristoyl‐glycero‐3‐phosphocholin and 1,3‐dimethylurate, supporting previous observations in PD *LRRK2* mutation carriers^10^ and indicating that caffeine absorption may be linked to PD.^35^, ^36^

Despite strong associations in our cohort, we were unable to correlate metabolite levels to typical measures of cognition such as MMSE or MoCA; likely a limitation of the early stage of disease in our cohort, where cognitive decline has yet to fully develop. This is supported by a significant association to MCI, which is more sensitive in early‐stage PD,^37^ but this does indicate that longitudinal studies of PD and cognitive decline are recommended to assess this further.

Given the significant increase of several metabolites in the same FAO pathway and supported by similar results in independent cohorts,^8^, ^9^ we conclude that FAO may be an important component in PD pathophysiology and the development of MCI. Moreover, our data suggest that FAO metabolites are potential biomarkers for the onset of PD and MCI, although further study is recommended.

## Author Roles

(1) Research Project: A. Conception, B. Organization, C. Execution; (2) Statistical Analysis: A. Design, B. Execution, C. Review and Critique; (3) Manuscript: A. Writing of the First Draft, B. Review and Critique.

F.B.: 1B, 1C

D.H.: 1B, 1C

A.P.: 1B, 1C

H.L.: 1B, 1C

S.N.: 1B. 1C

A.Y.: 1B, 1C, 3B

P.Y.W.M.: 1B, 1C, 3B

D.B.: 1B, 1C, 2C, 2B, 3B

M.S.K.: 1A, 1B, 2A, 2B, 2C, 3B

G.H.: 1A, 1B, 2A, 2B, 2C, 3A, 3B

## Full financial disclosures of all authors for the past 12 months

G.H. is a Parkinson's UK Senior Fellow (F‐1202). The research was supported by the National Institute for Health Research (NIHR) Newcastle Biomedical Research Unit based at Newcastle upon Tyne Hospitals NHS Foundation Trust and Newcastle University (D.B.). The research was also supported by NIHR Newcastle CRF Infrastructure funding (D.B.). PYWM is supported by a Clinician Scientist Fellowship Award (G1002570) from the Medical Research Council (UK), and also receives funding from Fight for Sight (UK), the UK National Institute of Health Research (NIHR) as part of the Rare Diseases Translational Research Collaboration, and the NIHR Biomedical Research Centre based at Moorfields Eye Hospital NHS Foundation Trust and UCL Institute of Ophthalmology. All authors have nothing further to disclose.

## Supporting information

Additional Supporting Information may be found in the online version of this article at the publisher's web‐site.

Supplementary Information
