# Supplementary material for: metabolic profiling of Parkinson's disease and mild cognitive impairment
Source: Mov Disord. 2017 Apr 10;32(6):927–32. doi: 10.1002/mds.26992 (PMC5485028; doi:10.1002/mds.26992)
Supplement: Supplementary file 1 — Supplementary Information [file MDS-32-927-s001.docx]

**Supplementary Material**

**Supplementary Table 1** – Phenotypic characteristics of cases and controls, showing mean age, age of onset, diagnosis (with standard deviation, SD), gender, mean mini-mental state examination (MMSE) score and Montreal Cognitive Assessment (MoCA) score. In addition mild cognitive impairment (MCI, stratified as yes, MCI-Y and no, MCI-N) is shown as percentage in cases and controls. P = significant difference by students t-test.

|  | ***Cases (n=41)*** | | ***Controls (n=40)*** | |  |
| --- | --- | --- | --- | --- | --- |
|  | ***Mean*** | ***SEM*** | ***Mean*** | ***SEM*** | ***P*** |
| ***Age (years)*** | 67.5 | 1.6 | 61.8 | 0.9 |  |
| ***Age of Onset (years)*** | 66.9 | 1.6 | - | - |  |
| ***Diagnosis (months)*** | 5.4 | 1.7 | - | - |  |
| ***Gender (M:F)*** | 28:13 | - | 20:20 | - |  |
|  |  |  |  |  |  |
| ***MMSE*** | 28.6 | 1.1 | 29.2 | 1.1 | 1.1x10^-02^ |
| ***MoCA*** | 25.3 |  | 27.0 | 2.5 | 2.5x10^-03^ |
|  |  |  |  |  |  |
| ***MCI-Y:N (%)*** | 62.5:37.5 | - | 0.0:100.0 | - | - |

**
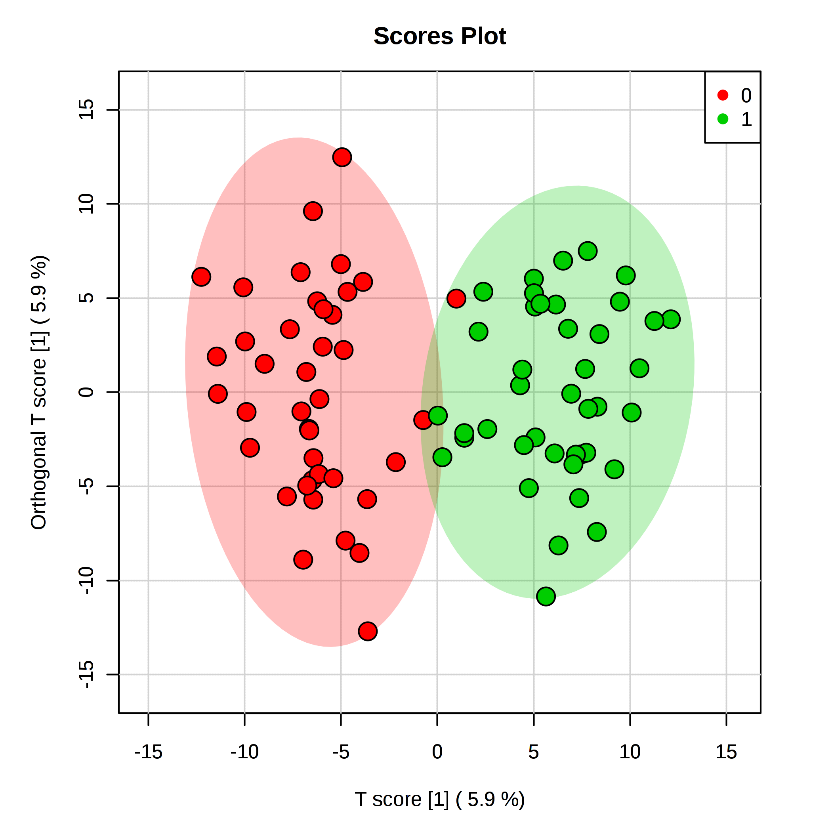
**

**Supplementary Figure 1 –** OPLS-DA plot of post-QC scaled metabolite data (n=1393) showing moderate separation of cases (red) and controls (green). Permutated OPLS-DA R^2^=0.77 and Q^2^=0.50 (n=2000 permutations).

**Supplementary Table 2** - Normalized mean metabolite levels in PD cases with mild-cognitive impairment (MCI-y) and without (MCI-n), showing standard error of the mean (SEM) and case/control comparison by *Mann-Whitney U testing.

| ***Compound*** | ***Canonical***  ***Pathway*** | ***Mean***  ***MCI-y (n=25)*** | ***SEM*** | ***Mean***  ***MCI-n (n=15)*** | ***SEM*** | ***Mann-***  ***Whitney U**** |
| --- | --- | --- | --- | --- | --- | --- |
| hexanoylglutamine | Fatty Acid Metabolism (Acyl Glutamine) | 2.38 | 0.33 | 1.14 | 0.15 | 2.0x10^-03^ |
| decanoylcarnitine | Fatty Acid Metabolism(Acyl Carnitine) | 2.13 | 0.22 | 1.34 | 0.10 | 5.0x10^-03^ |
| myristoleoylcarnitine | Fatty Acid Metabolism(Acyl Carnitine) | 1.90 | 0.20 | 1.38 | 0.08 | 4.4x10^-02^ |
| octanoylcarnitine | Fatty Acid Metabolism(Acyl Carnitine) | 1.29 | 0.08 | 0.99 | 0.04 | 1.8x10^-02^ |
| oleoylcarnitine | Fatty Acid Metabolism(Acyl Carnitine) | 1.60 | 0.12 | 1.18 | 0.06 | 1.0x10^-03^ |
| palmitoleoylcarnitine | Fatty Acid Metabolism(Acyl Carnitine) | 1.60 | 0.33 | 0.58 | 0.08 | 1.2x10^-03^ |
| suberoylcarnitine | Fatty Acid Metabolism(Acyl Carnitine) | 1.50 | 0.15 | 0.98 | 0.06 | 1.0x10^-03^ |
| octadecanedioate | Fatty Acid, Dicarboxylate | 1.70 | 0.25 | 0.94 | 0.13 | 6.3x10^-02^ |
| 3-hydroxysebacate | Fatty Acid, Monohydroxy | 2.29 | 0.36 | 1.44 | 0.19 | 5.0x10^-03^ |

**Supplementary Table 3 –** Statistical comparisons between the n= 20 significantly different metabolites Mini-Mental State Exam (MMSE) scores and Montreal Cognitive Assessment (MoCA) scores. *indicates comparison by Pearson’s correlation.

| ***Compound*** | ***Compound***  ***ID*** | ***Canonical Pathway*** | ***MMSE**** | ***MoCA**** |
| --- | --- | --- | --- | --- |
| oxalate (ethanedioate) | 20694 | Ascorbate and Aldarate Metabolism | 2.2x10^-01^ | 2.5x10^-01^ |
| tartronate (hydroxymalonate) | 20693 | Bacterial/Fungal | 2.8x10^-01^ | 3.2x10^-01^ |
| catechol sulfate | 35320 | Benzoate Metabolism | 3.7x10^-01^ | 3.6x10^-01^ |
|  |  |  |  |  |
| hexanoylglutamine | 54907 | Fatty Acid Metabolism (Acyl Glutamine) | 3.5x10^-01^ | 3.3x10^-01^ |
| decanoylcarnitine | 33941 | Fatty Acid Metabolism(Acyl Carnitine) | 1.7x10^-01^ | 6.8x10^-01^ |
| myristoleoylcarnitine | 48182 | Fatty Acid Metabolism(Acyl Carnitine) | 2.4x10^-01^ | 7.5x10^-01^ |
| octanoylcarnitine | 33936 | Fatty Acid Metabolism(Acyl Carnitine) | 8.8x10^-01^ | 7.5x10^-01^ |
| oleoylcarnitine | 35160 | Fatty Acid Metabolism(Acyl Carnitine) | 2.4x10^-01^ | 3.3x10^-01^ |
| palmitoleoylcarnitine | 53223 | Fatty Acid Metabolism(Acyl Carnitine) | 3.3x10^-01^ | 7.2x10^-01^ |
| suberoylcarnitine | 52990 | Fatty Acid Metabolism(Acyl Carnitine) | 4.6x10^-01^ | 9.9x10^-01^ |
| octadecanedioate | 36754 | Fatty Acid, Dicarboxylate | 9.8x10^-01^ | 7.2x10^-01^ |
| 3-hydroxysebacate | 31943 | Fatty Acid, Monohydroxy | 4.5x10^-01^ | 6.5x10^-01^ |
|  |  |  |  |  |
| 1-methylhistamine | 43831 | Histidine Metabolism | 2.6x10^-01^ | 3.7x10^-01^ |
| 1-myristoyl-GPC (14:0) | 45453 | Lysolipid | 5.6x10^-02^ | 7.3x10^-01^ |
| 2-myristoyl-GPC (14:0) | 35626 | Lysolipid | 5.4x10^-02^ | 9.7x10^-01^ |
| 1,3-dimethylurate | 32391 | Xanthine Metabolism | 3.5x10^-01^ | 2.6x10^-01^ |
|  |  |  |  |  |
| X - 12462 | 46620 | Unknown | 2.1x10^-01^ | 5.0x10^-01^ |
| X - 18249 | 46366 | Unknown | 7.2x10^-01^ | 5.0x10^-01^ |
| X - 21735 | 46904 | Unknown | 3.4x10^-01^ | 2.3x10^-01^ |
| X - 23756 | 49654 | Unknown | 6.2x10^-01^ | 7.7x10^-01^ |
